# Supplementary material for: Interaction of Impulsivity, Attention, and Intelligence in Early Adolescents Born Preterm without Sequelae
Source: Int J Environ Res Public Health. 2021 Aug 27;18(17):9043. doi: 10.3390/ijerph18179043 (PMC8431711; doi:10.3390/ijerph18179043)
Supplement: Supplementary file 1 [file ijerph-18-09043-s001.zip › ijerph-1300269-supplementary.pdf]

SUPPLEMENTARY MATERIAL:

S1. Average reached in the Intellectual Skills according to the Preterm Categories

Intellectual Skills	Preterm Categories	Mean	SD	Sig.	Effect
**Verbal Comprehension (VCI)**	Moderate Preterm	106.47	16.583	.791[a]	-.068
	Very Preterm	107.45	11.537		
**Visual-Spatial (VS)**	Moderate Preterm	99.00	19.433	.947[b]	-.007[d]
	Very Preterm	99.25	12.855		
**Fluid Reasoning (FRI)**	Moderate Preterm	92.49	14.190	.518[a]	-.301[c]
	Very Preterm	96.14	9.599		
**Working Memory (WMI)**	Moderate Preterm	100.51	15.806	.791[a]	-.069[c]
	Very Preterm	101.65	16.968		
**Processing Speed (PSI)**	Moderate Preterm	94.98	11.887	.923[a]	.012[c]
	Very Preterm	94.65	14.766		
**Intellectual Capacity (IQ)**	Moderate Preterm	100.08	20.902	.786[b]	-.024[d]
	Very Preterm	100.90	10.799		

Note. SD = Standard Deviation; [a] = Student's t-test; [b] = Mann-Whitney U-test; [c] = Cohen's d; [d] = Rosenthal r.

S2. Correlations between Intellectual and Attention Skills in Moderate Preterm

	**VCI**	**VSI**	**FRI**	**WMI**	**PSI**	**IQ**
**TOT**	.225^{a}	**.331**^{a}	**.414**^{a}	**.374**^{a}	**.419**^{a}	.275^{a}
Sig.	.121	.020	.009	.008	.003	.056
**CON**	.218^{b}	**.479**^{b}	**.577**^{b}	**.398**^{b}	**.555**^{b}	**.344**^{b}
Sig.	.132	.000	.000	.005	.000	.016
**AE**	**.407**^{a}	**.286**^{a}	**.523**^{a}	**.423**^{a}	**.544**^{a}	**.484**^{a}
Sig.	004	.047	.001	.002	.000	.000
**ICI**	.118^{b}	**.316**^{b}	.168^{b}	.213^{b}	.163^{b}	.275^{b}
Sig.	.419	.027	.307	.142	.262	.056

Note. TOT = Selective Attention; CON = Concentration Index; AE = Attentional Efficacy; ICI = Impulsivity Control Index; VCI = Verbal Comprehension Index; VSI = Visual-Spatial Index; FRI = Fluid Reasoning Index; WMI = Working Memory Index; PSI = Processing Speed Index; IQ = Total Intellectual Capacity; ^{a} = Pearson Correlation; ^{b} = Spearman Correlation. Significant data in bold.

S3. Correlations between Intellectual and Attention Skills in Very Preterm

	**VCI**	**VSI**	**FRI**	**WMI**	**PSI**	**IQ**
**TOT**	.120[a]	.406[a]	.301[a]	.167[a]	**.599[a]**	**.662[a]**
Sig.	.614	.076	.512	.483	.005	.001
**CON**	.095[a]	.439[a]	.229[a]	.125[a]	**.585[a]**	**.637[a]**
Sig.	.691	.053	.622	.600	.007	.003
**AE**	.171[a]	**.501[a]**	.375[a]	-.158[a]	**.581[a]**	**.532[a]**
Sig.	.472	.024	.407	.507	.007	.016
**ICI**	.046[b]	.314[b]	-.181[b]	-.182[b]	-.037[b]	.036[b]
Sig.	.846	.177	.698	.440	.877	.881

Note. TOT = Selective Attention; CON = Concentration Index; AE = Attentional Efficacy; ICI = Impulsivity Control Index; VCI = Verbal Comprehension Index; VSI = Visual-Spatial Index; FRI = Fluid Reasoning Index; WMI = Working Memory Index; PSI = Processing Speed Index; IQ = Total Intellectual Capacity; [a] = Pearson Correlation; [b] = Spearman Correlation. Significant data in bold.
